# Supplementary figures and images for: Evaluation of a global spring wheat panel for stripe rust: Resistance loci validation and novel resources identification
Source: PLoS One. 2019 Nov 13;14(11):e0222755. doi: 10.1371/journal.pone.0222755 (PMC6853611; doi:10.1371/journal.pone.0222755)

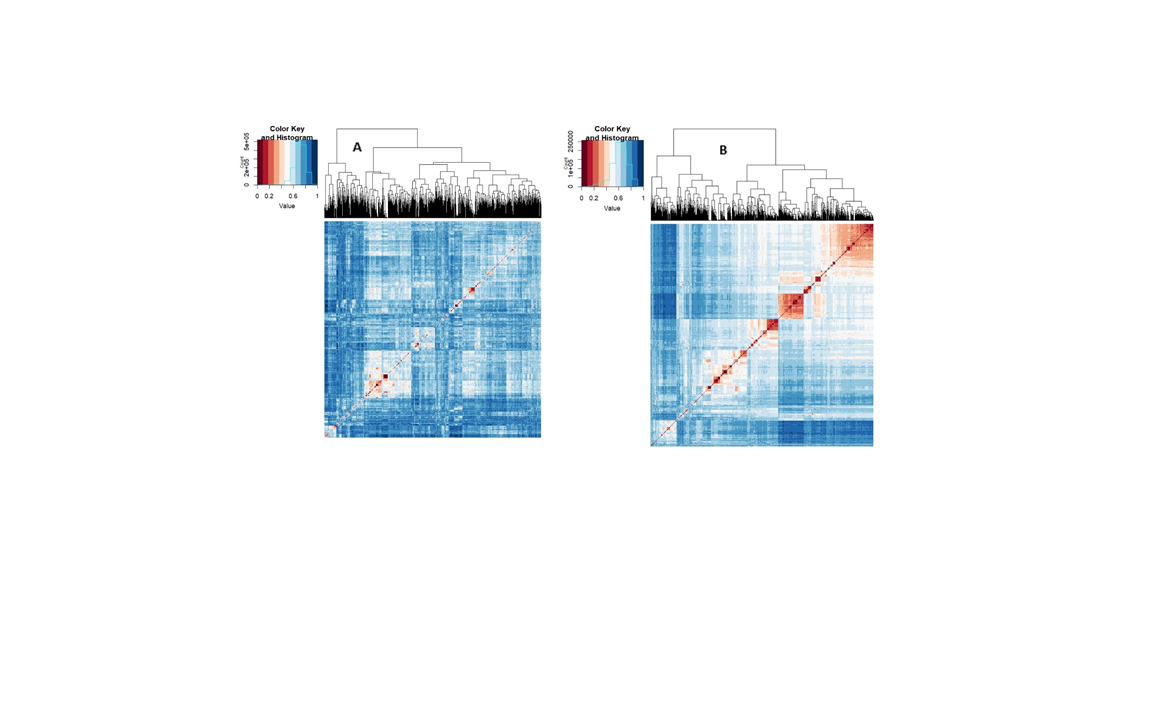

Supplement: S1 Fig — Heatmap estimated from the pairwise shared alleles for the improved accessions [A] and the landraces [B]. (TIF) [file pone.0222755.s003.tif]
