# Supplementary material for: Evaluation of a global spring wheat panel for stripe rust: Resistance loci validation and novel resources identification
Source: PLoS One. 2019 Nov 13;14(11):e0222755. doi: 10.1371/journal.pone.0222755 (PMC6853611; doi:10.1371/journal.pone.0222755)
Supplement: Attachment — Submitted filename: Response to Reviewers.docx [file pone.0222755.s004.docx]

6. Review Comments to the Author

Reviewer #1: 1) The cut points described in lines 196 - 198 are for single observations (R = 2, MR = 4, MS = 6 and S = 8). They don't take in to account average values. What happens if the average value is 5? Will it be MR or MS? That is why you need to describe your cut off point. On the same lines (355 - 358) you described Gimiza9 with average value of 4.6 as MS. I don't believe it's MS, rather it is closer to MR than MS. Describing the cut off value will avoid such confusions.

***Thank you for your comment, we added detailed description for the cutoff in the materials and methods as you suggested.***

2) Lines 201 - 202: The authors described how they calculated the ACI; but at no point did they use the ACI values in the results or discussions part. Hence, you may delete the description in the materials and methods part.

***ACI description was removed from the materials and methods***

3) The introduction part is still too long. It can be significantly shortened.

***Unnecessary parts were removed from the introduction***

Reviewer #2: Dear Dr Ibrahim Elbasyoni,
all comments have been addressed. Critical comments have been considered. Please change only line 107: "accession" to accessions.

***Thank you so much for your comment the required correction was conducted***

The manuscript provides now valuable information's about genotypes, molecular markers, usable for resistance breeding against yellow rust. The northern regions of Africa are effected by many abiotic and biotic stresses. Yellow rust epidemics have been observed in European countries as well, so that genotypes carrying effective resistances in this study are usable in the future as the basis for an increased resistance level of elite material.

***Thank you so much for the positive comment.***
